# Type-dependent action modes of *Tt*AA9E and *Ta*AA9A acting on cellulose and differently pretreated lignocellulosic substrates

**DOI:** 10.1186/s13068-017-0721-4

**Published:** 2017-02-22

**Authors:** In Jung Kim, Nari Seo, Hyun Joo An, Jae-Han Kim, Paul V. Harris, Kyoung Heon Kim

**Affiliations:** 1 Department of Biotechnology, Korea University, Graduate School, Seoul, 02841 South Korea; 2 Graduate School of Analytical Science & Technology and Asia-Pacific Glycomics Reference Site, Chungnam National University, Daejeon, 34134 South Korea; 3 Department of Food and Nutrition, Chungnam National University, Daejeon, 34134 South Korea; 4 Novozymes, Davis, CA 95618 USA

**Keywords:** Auxiliary activity family 9, Lytic polysaccharide monooxygenase, Glycoside hydrolase family 61, Cellulose oxidation, Synergism, Cellulose hydrolysis

## Abstract

**Background:**

Lytic polysaccharide monooxygenase (LPMO) is a group of recently identified proteins that catalyze oxidative cleavage of the glycosidic linkages of cellulose and other polysaccharides. By utilizing the oxidative mode of action, LPMOs are able to enhance the efficiency of cellulase in the hydrolysis of cellulose. Particularly, auxiliary activity family 9 (AA9) is a group of fungal LPMOs that show a type-dependent regioselectivity on cellulose in which Types 1, 2, and 3 hydroxylate at C1, C4, and C1 and C4 positions, respectively. In this study, we investigated comparative characteristics of *Tt*AA9E from *Thielavia terrestris* belonging to Type 1 and *Ta*AA9A from *Thermoascus aurantiacus* belonging to Type 3 on cellulose and pretreated lignocellulose.

**Results:**

From product analysis, *Tt*AA9E dominantly generated oligosaccharides with an aldonic acid form, which is an evidence of C1 oxidation, while *Ta*AA9A generated oligosaccharides with both aldonic acid and 4-ketoaldose forms, which is evidence of C1 and C4 oxidations, respectively. For hydrolysis of cellulose (Avicel) by cellulase, higher synergism was observed for *Tt*AA9E than for *Ta*AA9A. For hydrolysis of pretreated lignocellulose using rice straw, synergistic behaviors of *Tt*AA9E and *Ta*AA9A were different depending on the pretreatment of rice straw. Specifically, on acid-pretreated rice straw, *Tt*AA9E showed a higher synergism than *Ta*AA9A while on alkali-pretreated rice straw, *Ta*AA9A showed a higher synergism than *Tt*AA9E.

**Conclusions:**

We show type-dependent action modes of *Tt*AA9E and *Ta*AA9A for cellulose oxidation together with substrate-dependent synergistic hydrolysis of cellulosic substrates. The results obtained from this study indicate the different behaviors of AA9s on cellulose and pretreated lignocellulose, suggesting a selection of AA9 proteins specific to substrates is required for industrial utilization.

**Electronic supplementary material:**

The online version of this article (doi:10.1186/s13068-017-0721-4) contains supplementary material, which is available to authorized users.

## Background

Recently, the fortification of cellulase preparations by adding synergistic proteins was shown to be effective in facilitating the enzymatic saccharification of lignocellulose [1]. Synergistic proteins lack hydrolytic ability but play a significant role in promoting cellulase activity in the hydrolysis of lignocellulose [1–3]. Some of the synergistic proteins were reported to increase the accessibility of cellulose to cellulase through the modification or disruption of crystalline regions which are typically less inaccessible to cellulase [4]. Utilization of highly efficient synergistic proteins can reduce the total cellulase loadings required to achieve an industrially applicable cellulose conversion (i.e., 80–90%), thus reducing the cost for saccharification of lignocellulose [3].

There has been much attention on the lytic polysaccharide monooxygenases (LPMOs), a family of recently discovered synergistic proteins. The distinguishing feature of LPMOs is their unique oxidative cleavage action on polysaccharides [5–7]. Cellulose cleavage by LPMOs is known to involve the reduction of Cu^2+^ at the active site and the abstraction of H and hydroxylation [5, 6, 8]. Due to their oxidative mode of action, LPMOs require external electron donors such as ascorbic acid, reduced glutathione, gallate, or sodium azide for their activity [6, 7, 9]. In addition to these synthetic reducing agents, lignin, which naturally exists in lignocellulose, or enzymes such as cellobiose dehydrogenase (CDH) and glucose-methanol-choline oxidase/dehydrogenase (GMC) family of oxidoreductase can also act as reducing agents for LPMOs [8, 10–13]. LPMOs target the crystalline region of the cellulose surface which is typically more recalcitrant to cellulase action. This surface oxidation by LPMOs may induce structural modification of the recalcitrant cellulose, making it more amendable to subsequent hydrolysis by cellulase through the creation of additional chain ends [14, 15].

Family auxiliary activity 9 (AA9), previously known as glycoside hydrolase family 61 (GH61), is the fungal family of LPMOs active on cellulose [3, 7, 9, 16, 17]. Currently, AA9s are supplemented to some commercial cellulase preparations, where they have been shown to synergise with cellulase at industrially applicable levels of cellulose hydrolysis yields (i.e., 80–90%) [3]. Based on sequence similarity, AA9s are categorized into Types 1, 2, and 3, and each type recognizes different sites of cellulose for oxidation. Specifically, Types 1 and 2 predominantly utilize C1 (reducing end) and C4 (non-reducing end) oxidations, generating oxidized oligosaccharides in the form of aldonic acid and 4-ketoaldose, respectively [5, 8, 18, 19]. Type 3 catalyzes both C1 & C4 oxidations, generating both aldonic acid and 4-ketoaldose forms of cellooligosaccharides [18].

The molecular functions of Type 1 *Tt*AA9E from *Thielavia terrestris* and Type 3 *Ta*AA9A from *Thermoascus aurantiacus* were characterized earlier [3, 6, 20]. Although the direct oxidative action of *Ta*AA9A was already studied through product analysis, that of *Tt*AA9E has not been studied [6]. The structure of both AA9s was determined, and both were shown to improve the hydrolytic efficiency of lignocellulosic biomass by cellulase [3, 6]. Especially, the expression of *Ta*AA9A along with cellulase in a commercial cellulolytic strain of *Trichoderma reesei* enabled the reduction of cellulase loadings to a half of that required by a strain expressing only cellulase [3]. Furthermore, their oxidative actions were verified by the increase of synergistic activity of those AA9s in the presence of reducing cofactors such as gallate, lignin, ascorbic acid, or CDH in the hydrolysis of cellulose and pretreated lignocellulose [6, 11, 20]. With their high synergistic activity, *Tt*AA9E and *Ta*AA9A are industrially applicable.

In order to better understand the oxidative and synergistic activities of *Tt*AA9E and *Ta*AA9A, in this study, we have comparatively examined these two AA9s. First, reaction products which were directly generated from cellulose with AA9s were analyzed using matrix-assisted laser desorption/ionization-tandem time-of-flight mass spectrometry (MALDI-TOF/TOF MS) to investigate the oxidative mode of cleavage actions of *Tt*AA9E and *Ta*AA9A. Second, their synergistic activities with cellulase were studied against pure cellulose (Avicel) and rice straw samples using two different pretreatments (e.g., acid- and alkali-pretreatments as the two representative pretreatment methods). It is because pretreated lignocellulose varies in its composition and physical properties that could significantly affect the synergistic hydrolysis of AA9s [10–12]. Our comparative study of these two different AA9s in the oxidation of cellulose and the synergism with cellulase will shed light on the substrate specificities and functional variations of AA9s, which in turn will be helpful for the customization of AA9s in their industrial applications.

## Results and discussion

### Oxidative cleavage of cellulose by *Tt*AA9E and *Ta*AA9A

**Fig. 1:**
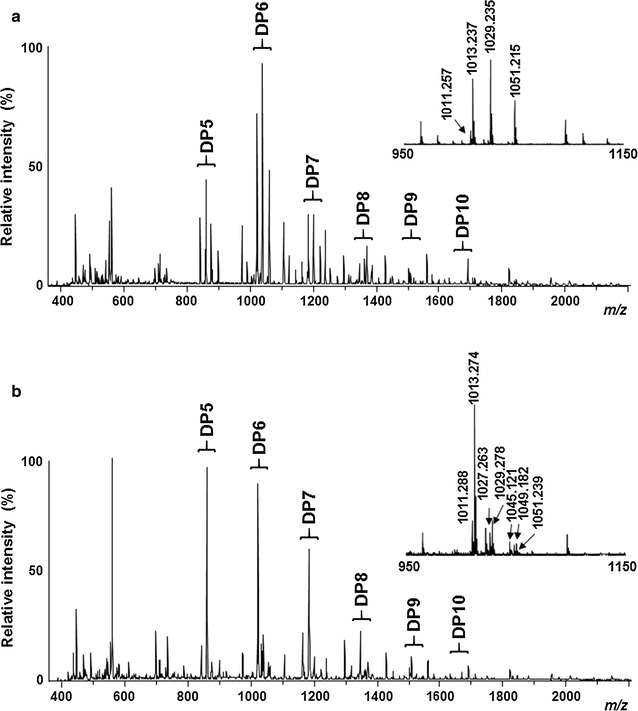
Product analysis obtained from the incubation of cellulose with *Tt*AA9E or *Ta*AA9A using MALDI-TOF/TOF MS. Avicel (5%, w/v) was incubated with *Tt*AA9E or *Ta*AA9A (1 mg/g Avicel) in 50 mM sodium acetate (pH 5.0) with 10 mM of ascorbic acid at 50 °C for 4 days. Overall profiles of products with sodium saturation generated from the incubation of cellulose with **a** *Tt*AA9E or **b** *Ta*AA9A in the presence of 10 mM ascorbic acid. Insets are expanded mass spectra for DP6 products obtained from the incubation of cellulose with **a** *Tt*AA9E or **b** *Ta*AA9A. 100% relative intensity represents 3.31 × 10^4^ and 2.62 × 10^4^ arbitrary units (a.u.) for the full spectra of *Tt*AA9E and *Ta*AA9A, respectively, and 3.31 × 10^4^ and 2.24 × 10^4^ a.u. for the close-up peaks of DP6 for *Tt*AA9E and *Ta*AA9A, respectively

To investigate the direct enzymatic activities of *Tt*AA9E and *Ta*AA9A towards cellulose, the reaction products from pure cellulose were analyzed by MALDI-TOF/TOF MS (Fig. 1). Our studies using Avicel revealed that both *Tt*AA9E and *Ta*AA9A cleave crystalline cellulose in the presence of 10 mM ascorbic acid to produce oxidized oligosaccharides with various degrees of polymerization (DPs). However, the two AA9s produce distinctively different product profiles (Fig. 1). These results indicate that both AA9s have the endo-type oxidative cleavage mode but at the same time different oxidative regioselectivities toward β-glycosidic linkages on cellulose.

**Fig. 2:**
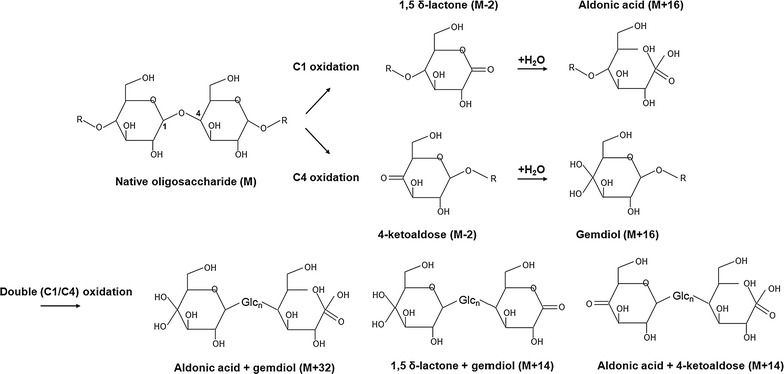
Scheme of oxidative cleavage reaction by LPMOs [18]. Possible products by C1 oxidation are aldonic acid (M+16, where M indicates the mass of native oligosaccharides), sodium adduct of aldonic acid (M+16-H+Na), and 1,5 δ-lactone (M−2), and those by C4 oxidation are 4-ketoaldose (M−2) and gemdiol (M+16). A product that contains both aldonic acid and gemdiol (M+32), 1,5 δ-lactone and gemdiol (M+14), or aldonic acid and 4-ketoaldose (M+14) is an evidence of double (C1/C4) oxidation

To verify their oxidative regioselectivities, oxidized sugars were identified by mass-based prediction of the possible products based on a previous report (inset in Figs. 1, 2) [19]. Possible products of C1 oxidation by LPMOs can be aldonic acid (M+16, where M indicates the mass of native oligosaccharides), sodium adduct of aldonic acid (M+16-H+Na), and 1,5 δ-lactone (M−2) whereas 4-ketoaldose (M−2) and gemdiol (M+16) are generated by C4 oxidation. In the case of C1&C4 oxidations, all of the above-listed sugars and doubly oxidized sugars containing both aldonic acid and gemdiol (M+32), 1,5 δ-lactone and gemdiol (M+14), or aldonic acid and 4-ketoaldose (M+14) can be observed (Fig. 2) [19]. In this study, *Tt*AA9E, whose products have not been reported in the literature, dominantly produced aldonic acid forms of oligosaccharides (Fig. 1a). The peak corresponding to M-2 is likely to be the unstable lactone form of aldonic acid rather than 4-ketoaldose, considering the exclusive formation of aldonic acid and its sodium adduct by the action of *Tt*AA9E. In addition, double oxidized sugar was not observed with *Tt*AA9E, which argues against C1 and C4 oxidations. These results indicate the strict C1 oxidative mode of *Tt*AA9E. In this study, *Ta*AA9A showed a very low cleaving activity on Avicel, and its full spectrum was difficult to distinguish from that of the control, which was incubated without *Ta*AA9A (data not shown). Nevertheless, the masses corresponding to possible non-reducing end-oxidized and doubly oxidized sugars (M-2, M+14, and M+32) and aldonic acid forms were obtained with *Ta*AA9A, which were not observed with the control (Fig. 1b; Additional file 1: Figure S1).

Although the peak corresponding to M-2 could indicate either reducing end oxidation (1,5 δ-lactone of aldonic acid) or non-reducing end oxidation (4-ketoaldose), doubly oxidized sugar (M+14 and M+32) ensures the occurrence of oxidations both at reducing and non-reducing ends by *Ta*AA9A, something not observed with the strict C1-oxidizing *Tt*AA9E. This is consistent with the previous study with *Ta*AA9A, in which *Ta*AA9A was shown to oxidize at both reducing and nonreducing ends of phosphoric acid-swollen cellulose (PASC) [6]. Thus, *Ta*AA9A was considered to use the C1 and C4 oxidation mode in this study.

AA9s are known to possess a type-dependent selective mode of action for oxidation of cellulose: Type 1 dominantly oxidizes C1, Type 2 dominantly oxidizes C4, and Type 3 can oxidize both C1 and C4 [18]. This close correlation between the sequence/structure-based classification and the oxidation mode agrees with our results from the product analysis of *Tt*AA9E and *Ta*AA9A. Specifically, *Tt*AA9E belonging to Type 1 induces an oxidative cleavage of β-1,4-glycosidic bonds of cellulose via C1 oxidation while *Ta*AA9A belonging to Type 3 cleaves the β-1,4-glycosidic bonds via both C1 and C4 oxidations. The generation of oxidized products by *Tt*AA9E was much higher compared to that by *Ta*AA9A (Additional file 1: Figure S1). Through the cleavage action of *Tt*AA9E, *Tt*AA9E was shown to have a higher PMO activity.

### Synergistic activity of *Tt*AA9E and *Ta*AA9A on the enzymatic hydrolysis of cellulose

**Fig. 3:**
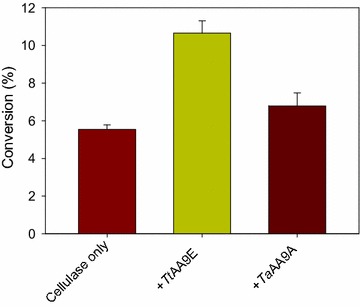
Synergistic activities of *Tt*AA9E and *Ta*AA9A in the hydrolysis of cellulose by cellulase. Avicel (1%, w/v) was hydrolyzed using Celluclast 1.5 L (0.9 FPU/g Avicel) without (control), with *Tt*AA9E, or with *Ta*AA9A (1 mg/g Avicel) in 50 mM sodium acetate buffer (pH 5.0) containing NaN_3_ at 50 °C for 48 h. The reducing sugar was determined by DNS assay, and the reducing sugar yields were expressed as % of the theoretical maximal yield of glucose. The data represent means ± standard errors obtained from the three experimental replicates

From the product analysis earlier in this study, different modes of action were observed for the two AA9s when they were directly applied to cellulose (Fig. 1). In this study, furthermore, the synergistic effects of *Tt*AA9E and *Ta*AA9A on the enzymatic hydrolysis of pure cellulose were investigated (Fig. 3). Avicel was hydrolyzed by a commercial cellulase preparation, Celluclast 1.5 L either with or without AA9s in the presence of antibiotic NaN_3_. In contrast to a previous study of *Tt*AA9E and *Ta*AA9A [3], which reported no synergistic activity of these two AA9s on pure cellulose without adding reducing agents, the two enzymes showed synergism with cellulase in this study. This is possibly because the antibiotic NaN_3_ in the reaction mixture was also able to work as the reducing power for AA9s activities as described in an earlier study [9]. The synergistic activity on cellulose was higher for *Tt*AA9E than for *Ta*AA9A. At a cellulase loading of 0.9 FPU/g Avicel, the reducing sugar yields with *Tt*AA9E and *Ta*AA9A were 1.9 and 1.2 fold higher than the control without AA9s in the hydrolysis, respectively (*p* < 0.05). The higher synergistic activity of *Tt*AA9E was consistent with its higher cleavage activity observed in the product analysis earlier in this study.

Even with the experimentally determined relation between AA9 activity on cellulose and the synergism with cellulase in this study, it is difficult to generalize how type-dependent regioselectivity is associated with the synergistic activity of AA9s with cellulase. Only a few AA9s such as *Tt*AA9E, *Ta*AA9A, *Pc*GH61D, *St*Cel61a, and *Cg*AA9 have been studied for their synergistic activities [3, 9, 20, 21]. In addition, those studies on synergism were performed under various enzymatic reaction conditions such as different loadings of cellulase and AA9, different types and concentrations of reducing cofactors and cellulosic substrates and so on, which might have highly affected synergistic characteristics [1].

### Synergistic activity of *Tt*AA9E and *Ta*AA9A on the enzymatic hydrolysis of pretreated lignocellulose

**Fig. 4:**
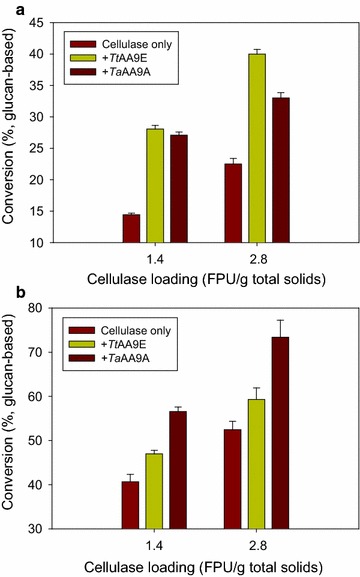
Synergistic activity of *Tt*AA9E or *Ta*AA9A in the hydrolysis of two types of pretreated rice straw by cellulase. A solid loading of 5% (w/v) of pretreated rice straw of **a** ACID or **b** ALKALI was hydrolyzed using Celluclast 1.5 L (1.4 and 2.8 FPU/g total solids) in the absence or presence of *Tt*AA9E or *Ta*AA9A (5 mg/g cellulose) in 50 mM sodium acetate buffer (pH 5.0) in the presence of 0.02% of NaN_3_ and 1 mM of MnSO_4_ at 50 °C for 8 days. The reducing sugar was determined by DNS assay, and the reducing sugar yields were expressed as % of the theoretical maximal yield of glucose. The data represent means ± standard errors obtained from the three experimental replicates

Depending on the lignocellulose pretreatment method used, pretreated lignocellulosic substrates have significantly different compositions and physical properties as a result of the pretreatment. The resulting composition and physical properties affect the mode of action of enzymatic hydrolysis of the pretreated lignocellulose, by which enzymes target and hydrolyze the pretreated lignocellulose [22–24]. Indeed, AA9s have been reported to display different behaviors against different pretreated lignocellulosic substrates [10–12]. Therefore, following the synergistic study on cellulose, comparison was made for the synergism of *Tt*AA9E and *Ta*AA9A in the enzymatic hydrolysis of pretreated lignocellulose (Fig. 4) although these two AA9s had been already assessed for their synergistic activities on other pretreated lignocellulose such as acid-pretreated corn stover or steam-exploded birch before [3, 25]. Two types of rice straw pretreated using dilute sulfuric acid (ACID) and aqueous ammonia (ALKALI), which had different compositions mainly in terms of cellulose, hemicellulose, and lignin, were generated in this study [26]. As described in Methods, ACID had a lower content of hemicellulose, but the lignin content in ACID was higher than that in the untreated rice straw. In contrast, ALKALI had a lower content of lignin than ACID.

Both *Tt*AA9E and *Ta*AA9A showed a synergistic activity in the enzymatic hydrolysis of ACID and ALKALI in the presence of cellulase at 1.4 and 2.8 FPU/g total solids (Fig. 4). As for *Ta*AA9A, regardless of its very low cleavage activity as shown in Fig. 1, its synergistic effect was exhibited with the pretreated rice straw. This could be because *Ta*AA9A might have induced the surface disruption of the pretreated rice straw substrates despite of the fact that *Ta*AA9A produced almost undetectable amounts of sugar products [4]. This disruption of the pretreated rice straw substrates by *Ta*AA9A could have been led to the facilitation of the hydrolysis of pretreated rice straws by cellulase.

*Tt*AA9E (1.9 or 1.8 fold) showed a higher synergistic activity than *Ta*AA9A (1.9 or 1.5 fold) on ACID, whereas the opposite results were obtained on ALKALI (1.1 or 1.2 fold in case of *Tt*AA9E and 1.4 fold in case of *Ta*AA9A) with cellulase loading of 1.4 or 2.8 FPU/g total solids, respectively. These results indicate that the synergistic activity of AA9s is specific for pretreatment types and is highly affected by the nature of the pretreated lignocellulose, which is often represented by its composition and physicochemical properties [10–12]. Therefore, to obtain higher hydrolysis yields, the employment of AA9s needs to be customized for the types of pretreatment and lignocellulose.

## Conclusions

In this study, we have performed a comparative study on the cellulose oxidative and synergistic activities of *Tt*AA9E and *Ta*AA9A, which are phylogenically Types 1 and 3 AA9s, respectively. The characterization of *Tt*AA9E and *Ta*AA9A on cellulose revealed that their regioselectivity on β-1,4-glycosidic bonds depends on their type. Aldonic acid derived from C1 oxidation was produced by the Type 1 AA9 (in case of *Tt*AA9E), and aldonic acid and 4-ketoaldose derived from C1 and C4 oxidations were produced by the Type 3 AA9 (in the case of *Ta*AA9A). In addition, these oxidative cleaving activities on cellulose were higher in *Tt*AA9E than in *Ta*AA9A. *Tt*AA9E and *Ta*AA9A exhibited different behaviors with regard to their synergistic activity with cellulase in the hydrolysis of both cellulose and pretreated lignocellulose. *Tt*AA9E showed a higher synergistic activity on cellulose, which was consistent with its higher cleaving activity observed from the product analysis of direct actions of the AA9s on cellulose. In summary, different functional properties of the two distinctive AA9s were demonstrated using cellulose and pretreated lignocellulose as substrates. Our results suggest that the systematic customization of AA9s depending on the pretreatment types and biomass is necessary to maximize the hydrolysis efficiency and performance of AA9s.

## Methods

### AA9s and cellulase and pretreated rice straw

*Tt*AA9E and *Ta*AA9A used in this study were provided by Novozymes (Bagsvaerd, Denmark). For the assay of synergistic activity in the enzymatic hydrolysis of cellulose and pretreated lignocellulose, Celluclast 1.5 L (Novozymes), a commercial cellulase preparation produced by *T. reesei*, was used.

Two types of rice straw pretreated using dilute sulfuric acid and aqueous ammonia were used in this study, which were designated as ACID and ALKALI, respectively. The same pretreated substrates that were previously reported were applied for the synergistic study here [26]. ACID was prepared by using 1% (w/v) sulfuric acid at 190 °C with ramping and holding times for 3 min and 90 s, respectively, in a microwave digestion system. ALKALI was prepared by soaking in 14% (w/v) aqueous ammonia at 69 °C for 10 h. Then, both pretreated substrates were washed with water and dried at 45 °C [27, 28]. The composition of cellulose, hemicellulose, and lignin in ACID and ALKALI is also described in that study [26]. In brief, the contents of cellulose, hemicellulose, and lignin in untreated rice straw were 36.4 and 18.0, and 18.1% (w/w), respectively. In ACID, the contents of cellulose and lignin were 56.9 and 24.8% (w/w), respectively, while those of hemicellulosic components such as xylan, galactan, arabinan, and mannan were not determined since most of them were solubilized by acidic treatment. In ALKALI, lignin was substantially removed by alkaline treatment leading to the contents of cellulose, hemicellulose, and lignin being 47.6, and 16.1, and 15.1% (w/w), respectively.

### Analysis of enzymatic reaction products by MALDI-TOF/TOF MS

Enzymatic reactions were performed by incubating 5% (w/v) of Avicel PH101 (Sigma-Aldrich, St. Louis, MO) with 1 mg of *Tt*AA9E or *Ta*AA9A/g Avicel in 50 mM sodium acetate (pH 5.0) at 50 °C for 4 days. After centrifugation of the reaction mixture, the supernatant containing soluble products was taken for analysis by MALDI-TOF/TOF MS. Prior to the injection into MALDI-TOF/TOF MS, sample was prepared as follows. After purification and desalting of the reaction product, 1 μL of the product dissolved in water was spotted onto a stainless steel target plate. Next, sodium saturation was achieved using the solutions including 0.3 μL of 0.01 M NaCl followed by the addition of 0.5 μL of 50 mg/mL 2,5-dihydroxybenzoic acid in 50% (w/v) acetonitrile. Then, the spot solution was subjected to vacuum drying for homogeneous crystallization. An ultrafleXtreme system (Bruker Daltonics, Billerica, MA) with a positive ion reflectron mode was used for MALDI-TOF/TOF MS analysis. The detailed analytical methods were presented in a previous study [29]. The calibration of mass spectra was externally carried out using cellooligosaccharides as standards. Processing of raw MS data was performed using a FlexAnalysis software (version 3.3; Bruker Daltonics). Using a signal-to-noise ratio of 3.0, MS peaks were filtered, followed by deconvolution.

### Synergistic hydrolysis by cellulase with *Tt*AA9E or *Ta*AA9A

For the hydrolysis of cellulose, 1% (w/v) of Avicel PH-101 was incubated with 0.9 FPU/g Avicel of Celluclast 1.5 L in the presence or absence of 1 mg of *Tt*AA9E or *Ta*AA9A/g Avicel in 50 mM sodium acetate buffer (pH 5.0) at 50 °C for 2 days. For the hydrolysis of pretreated rice straw, 5% (w/v) of ACID or ALKALI was incubated with Celluclast 1.5 L (1.4 and 2.8 FPU/g total solids) in the presence or absence of 5 mg of *Tt*AA9E or *Ta*AA9A/g cellulose in 50 mM sodium acetate buffer (pH 5.0) containing 1 mM MnSO_4_ at 50 °C for 8 days. Sodium azide at 0.02% (w/v) was added to all the synergistic reactions as an antibiotic or reducing agent. After the reaction, the reaction mixture was boiled at 95 °C for 5 min to terminate the reaction, and subjected to centrifugation at 30,000×*g* for 5 min. Finally, reducing sugars in the supernatant were quantified by the 3,5-dinitrosalicylic acid (DNS) assay at 540 nm using d-glucose as the standard. The extent of synergism is represented as the fold increase as follows. Extent of synergism = Reducing sugar yield from the hydrolysis of *Tt*AA9E or *Ta*AA9A with cellulase/Reducing sugar yield from the hydrolysis of cellulase only.

## Additional file

**Additional file 1: Figure S1.** Expanded mass spectra of reaction products obtained from the incubation of cellulose with *Tt*AA9E or *Ta*AA9A using MALDI-TOF/TOF MS. Avicel (5%, w/v) was incubated with *Tt*AA9E or *Ta*AA9A (1 mg/g Avicel) in 50 mM sodium acetate (pH 5.0) with 10 mM of ascorbic acid at 50 °C for 4 days. Expanded mass spectra for **a** DP5, **b** DP6, and **c** DP7 products obtained from the incubation of cellulose with *Tt*AA9E or *Ta*AA9A. 100% relative intensity represents 4.62 × 10^4^ a.u. for DP5 and DP6 and 3.00 × 10^4^ a.u. for DP7. Possible products by C1 oxidation are aldonic acid, sodium adduct of aldonic acid, and 1,5 δ-lactone, and those by C4 oxidation are 4-ketoaldose and gemdiol. Product that contain both aldonic acid and gemdiol, 1,5 δ-lactone and gemdiol, or aldonic acid and 4-ketoaldose is an evidence of double (C1/C4) oxidation. The detailed mass information is shown in the text and Fig. 2.

## Abbreviations

LPMO: lytic polysaccharide monooxygenase
AA9: auxiliary activity 9
CDH: cellobiose dehydrogenase
GH61: glycoside hydrolase family 61
MALDI-TOF/TOF MS: matrix-assisted laser desorption/ionization tandem time-of-flight mass spectrometry
DP: degree of polymerization
DNS: 3,5-dinitrosalicylic acid
FPU: filter paper unit
