# Supplementary material for: Type-dependent action modes of TtAA9E and TaAA9A acting on cellulose and differently pretreated lignocellulosic substrates
Source: Biotechnol Biofuels. 2017 Feb 22;10:46. doi: 10.1186/s13068-017-0721-4 (PMC5322634; doi:10.1186/s13068-017-0721-4)
Supplement: Supplementary file 1 — Additional file 1: Figure S1. Expanded mass spectra of reaction products obtained from the incubation of cellulose with TtAA9E or TaAA9A using MALDI-TOF/TOF MS. Avicel (5%, w/v) was incubated with TtAA9E or TaAA9A (1 mg/g Avicel) in 50 mM sodium acetate (pH 5.0) with 10 mM of ascorbic acid at 50 °C for 4 days. Expanded mass spectra for a DP5, b DP6, and c DP7 products obtained from the incubation of cellulose with TtAA9E or TaAA9A. 100% relative intensity represents 4.62 × 10^4^ a.u. for DP5 and DP6 and 3.00 × 10^4^ a.u. for DP7. Possible products by C1 oxidation are aldonic acid, sodium adduct of aldonic acid, and 1,5 δ-lactone, and those by C4 oxidation are 4-ketoaldose and gemdiol. Product that contain both aldonic acid and gemdiol, 1,5 δ-lactone and gemdiol, or aldonic acid and 4-ketoaldose is an evidence of double (C1/C4) oxidation. The detailed mass information is shown in the text and Fig. 2. [file 13068_2017_721_MOESM1_ESM.doc]

**Additional Fig. 1**

**
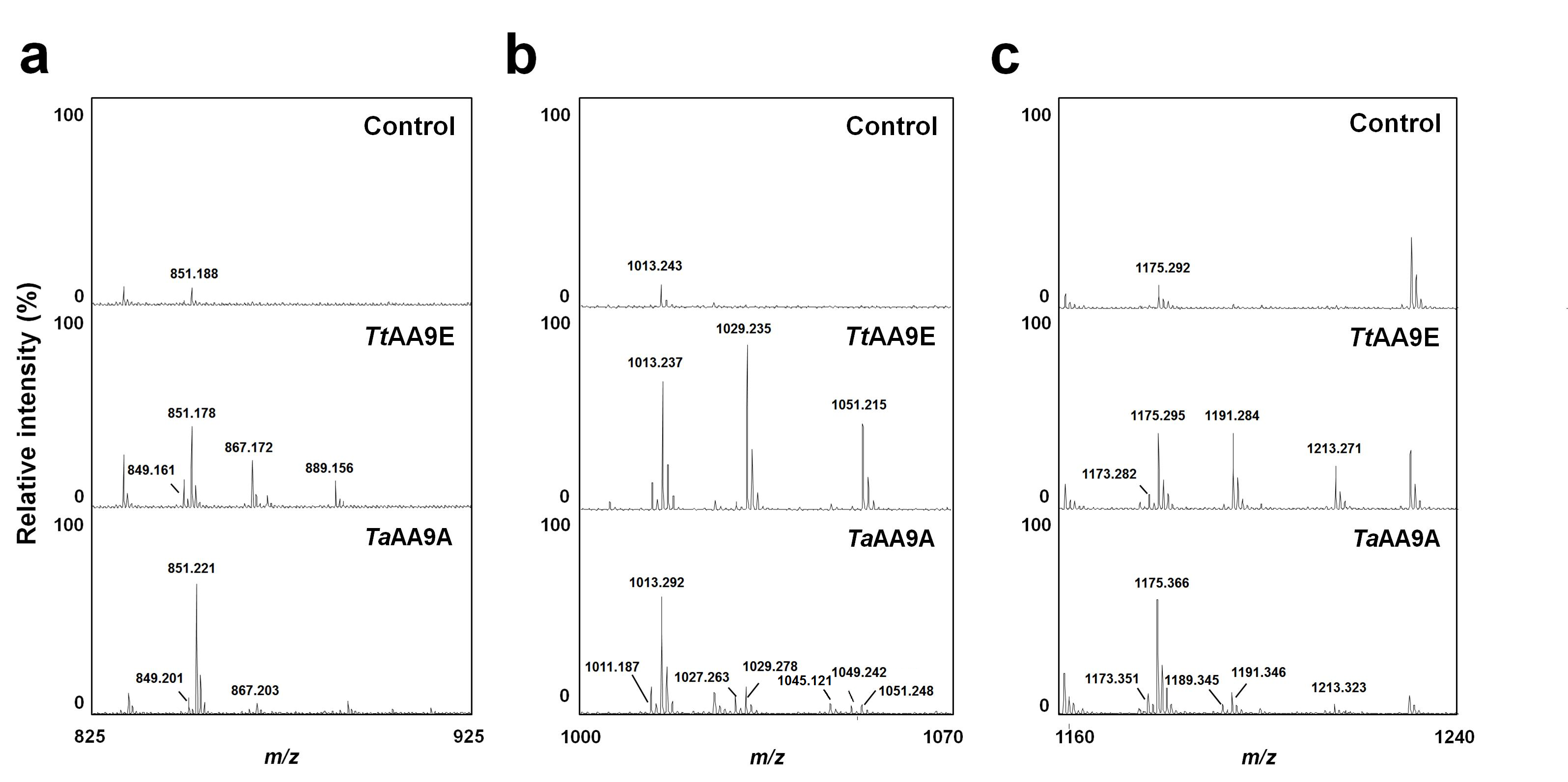
**

**Additional file 1: Figure S1** Expanded mass spectra of reaction products obtained from the incubation of cellulose with *Tt*AA9E or *Ta*AA9A using MALDI-TOF/TOF MS**.** Avicel (5%, w/v) was incubated with *Tt*AA9E or *Ta*AA9A (1 mg/g Avicel) in 50 mM sodium acetate (pH 5.0) with 10 mM of ascorbic acid at 50°C for 4 days. Expanded mass spectra for **a** DP5, **b** DP6, and **c** DP7 products obtained from the incubation of cellulose with *Tt*AA9E or *Ta*AA9A. 100% relative intensity represents 4.62 x 10^4^ a.u. for DP5 and DP6 and 3.00 x 10^4^ a.u. for DP7. Possible products by C1 oxidation are aldonic acid, sodium adduct of aldonic acid, and 1,5 δ-lactone, and those by C4 oxidation are 4-ketoaldose and gemdiol. Product that contain both aldonic acid and gemdiol, 1,5 δ-lactone and gemdiol, or aldonic acid and 4-ketoaldose is an evidence of double (C1/C4) oxidation. Their mass information is described in the text. The detailed mass information is shown in the text and Fig. 2
